# Induction of Fc-Mediated Effector Functions Against a Stabilized Inner Domain of HIV-1 gp120 Designed to Selectively Harbor the A32 Epitope Region

**DOI:** 10.3389/fimmu.2019.00677

**Published:** 2019-04-02

**Authors:** Maria L. Visciano, Neelakshi Gohain, Rebekah Sherburn, Chiara Orlandi, Robin Flinko, Amir Dashti, George K. Lewis, William D. Tolbert, Marzena Pazgier

**Affiliations:** Division of Vaccine Research of Institute of Human Virology, University of Maryland School of Medicine, Baltimore, MD, United States

**Keywords:** HIV envelope, ID (Inner domain) immunogen, ADCC (Antibody dependent cellular cytotoxicity), A32 epitope, Fc-mediated effector function

## Abstract

Recent clinical trials and studies using nonhuman primates (NHPs) suggest that antibody-mediated protection against HIV-1 will require α-HIV envelope humoral immunity beyond direct neutralization to include Fc-receptor (FcR) mediated effector functions such as antibody-dependent cellular cytotoxicity (ADCC). There is also strong evidence indicating that the most potent ADCC response in humans is directed toward transitional non-neutralizing epitopes associated with the gp41-interactive face of gp120, particularly those within the first and second constant (C1–C2) region (A32-like epitopes). These epitopes were shown to be major targets of ADCC responses during natural infection and have been implicated in vaccine-induced protective immunity. Here we describe the immunogenicity of ID2, an immunogen consisting of the inner domain of the clade A/E 93TH057 HIV-1 gp120 expressed independently of the outer domain (OD) and stabilized in the CD4-bound conformation to harbor conformational A32 region epitopes within a minimal structural unit of HIV-1 Env. ID2 induced A32-specific antibody responses in BALB/c mice when injected alone or in the presence of the adjuvants Alum or GLA-SE. Low α-ID2 titers were detected in mice immunized with ID2 alone whereas robust responses were observed with ID2 plus adjuvant, with the greatest ID2 and A32-specific titers observed in the GLA-SE group. Only sera from groups immunized in the presence of GLA-SE were capable of mediating significant ADCC using NKr cells sensitized with recombinant BaL gp120 as targets and human PBMCs as effectors. A neutralization response to a tier 2 virus was not observed. Altogether, our studies demonstrate that ID2 is highly immunogenic and elicits A32-specific ADCC responses in an animal host. The ID2 immunogen has significant translational value as it can be used in challenge studies to evaluate the role of non-neutralizing antibodies directed at the A32 subregion in HIV-1 protection.

## Introduction

The design of immunogens which induce broadly protective antibody responses against human immunodeficiency virus type 1 (HIV-1) is a major goal of HIV-1 vaccine development. This goal is formidable as HIV-1 evades immune surveillance via a number of escape mechanisms (1–3). Over the last few years neutralizing humoral responses have been observed to overcome some of these obstacles and provide protection in a subpopulation of chronically infected individuals (1, 4–9). Despite the significant progress in identification and characterization of broadly neutralizing antibodies (bnAbs) there are still multiple, challenging obstacles in the design of a successful candidate immunogen which, when coupled with appropriate immunization strategies, can induce effective neutralizing responses *in vivo* (10). These challenges are primarily linked to the unusual structural features associated bnAbs; such as the long complementary determining region 3 (CDR H3) and the high level of somatic mutation of the variable (V) domain. The frequency of B cells for these unusual antibodies is very low and the time required for their full development from progenitors is remarkably long (11), making them very complex candidates for vaccine design.

By contrast, less is known about mechanisms of vaccine induced humoral responses that act solely through Fc-mediated effector functions, including antibody-dependent cell-mediated cytotoxicity (ADCC). Epitopes involved solely in Fc-mediated processes are usually exposed late during viral entry and are thus targeted by antibodies that lack direct neutralizing activity. One group of these potent ADCC targets constitute the CD4-inducible (CD4i) epitopes within the gp120 molecule, referred to as Cluster A epitopes (12–16). These epitopes become exposed on the target cell surface during viral entry after envelope trimers engage the host CD4 receptor and they persist on newly infected cell surfaces for extended periods of time (17–20), reviewed in (21–23). They are also expressed at the surface of infected cells, but only in cell populations that retain some levels of the CD4 receptor which is required for triggering envelope trimers on budding virions (15, 16, 23). We recently isolated and characterized, at the molecular level, the complexes of CD4-triggered gp120 with a number of monoclonal antibodies (mAbs) known to be capable of potent Fc-receptor mediated function from memory B cells of HIV-1 infected individuals that recognize the A32-like epitope within the Cluster A epitope region (14, 24, 25). Based on these studies, we mapped the A32 epitope into the highly conserved constant regions 1 and 2 (C1–C2) of the gp120 inner domain in the CD4-bound conformation. We also found that A32-like antibodies differ significantly from those involved in neutralization as they mostly possess moderate length CDR H3 loops and low degrees of V affinity maturation and therefore bypass the frequently observed somatic hypermutation hurdle in eliciting a protective antibody response (12, 26, 27). The high sequence conservation of the A32 epitope among different HIV isolates indicates the possibility that ADCC responses specific for this epitope region may be cross-reactive and multiple strains would therefore undergo limited immune escape. Indeed, the recent vaccination strategy tested in the RV144 vaccine trial partially confirmed these predictions. A32-like responses were induced with the RV144 vaccine and ADCC responses directed to the A32 epitope region were implicated in its protective effect (28). In the absence of IgA responses, ADCC correlated with a reduced infection risk (29, 30) with a very narrow array of antibody specificities involved in the protective effect. RV144 ADCC specificities included the linear epitopes in the V2 loop region (31) and the CD4-inducible conformational epitopes within the A32 region (32, 33), confirmed by blocking the plasma ADCC activity with the A32 Fab (32). Furthermore, most ADCC mAbs (19 of 23) isolated from vaccine recipients targeted multiple related but distinct conformational epitopes in the A32 region (31, 32). These antibodies displayed low levels of V_H_ chain somatic mutation (0.5–1.5%) and mediated cross-clade ADCC activity; clade B and CRF01 AE, as well as clade C, which was not represented in the vaccine (32); a canarypox ALVAC prime with the E.92TH023 gp120 membrane anchored insert and an AIDSVAX B/E gp120 boost.

Here we describe the immunogenicity of a gp120 sub domain immunogen, referred to as ID2, designed by our group to stimulate humoral responses involving solely FcR-effector mechanisms designed to elicit an ADCC response in the absence of a neutralizing response (34). ID2 consists of the inner domain (ID) of the clade A/E HIV-1 gp120 93TH057 isolate and was made to confer the minimal structural unit of gp120 stably presenting the non-neutralizing epitopes in the A32 region without any other known epitopes present (34). When injected into BALB/c mice, ID2 was able to elicit cross-clade A32-like antibody responses with ADCC activities against gp120_BaL_ coated cells.

## Materials and Methods

### ID2 Immunogen Expression and Purification

A HEK 293 cell line stably expressing the ID2 immunogen was generated using the plasmid previously used for transient protein production, as in (34). Freestyle 293 medium (Gibco) from cells grown for 6–7 days (8% CO2 at 37°C in shaker flasks rotating at 145 rpm) after inoculation (1 × 10^6^ cells/ml) was collected and passed through a 0.45 μm filter. ID2 was purified from the media using an N5-i5 IgG affinity column, which was made by coupling N5-i5 IgG to protein A resin using the Pierce protein A IgG plus orientation kit (Thermo Fisher Sci.). Media was passed over the column after equilibration in phosphate buffered saline (PBS) pH 7.2. The column was washed with 5–10 column volumes of PBS pH 7.2 and ID2 protein eluted with 0.1 M glycine pH 3.0. Elution fractions were concentrated and dialyzed against PBS pH 7.2 prior to use in animal studies.

### Immunization and Blood Collections

**Figure 1:**
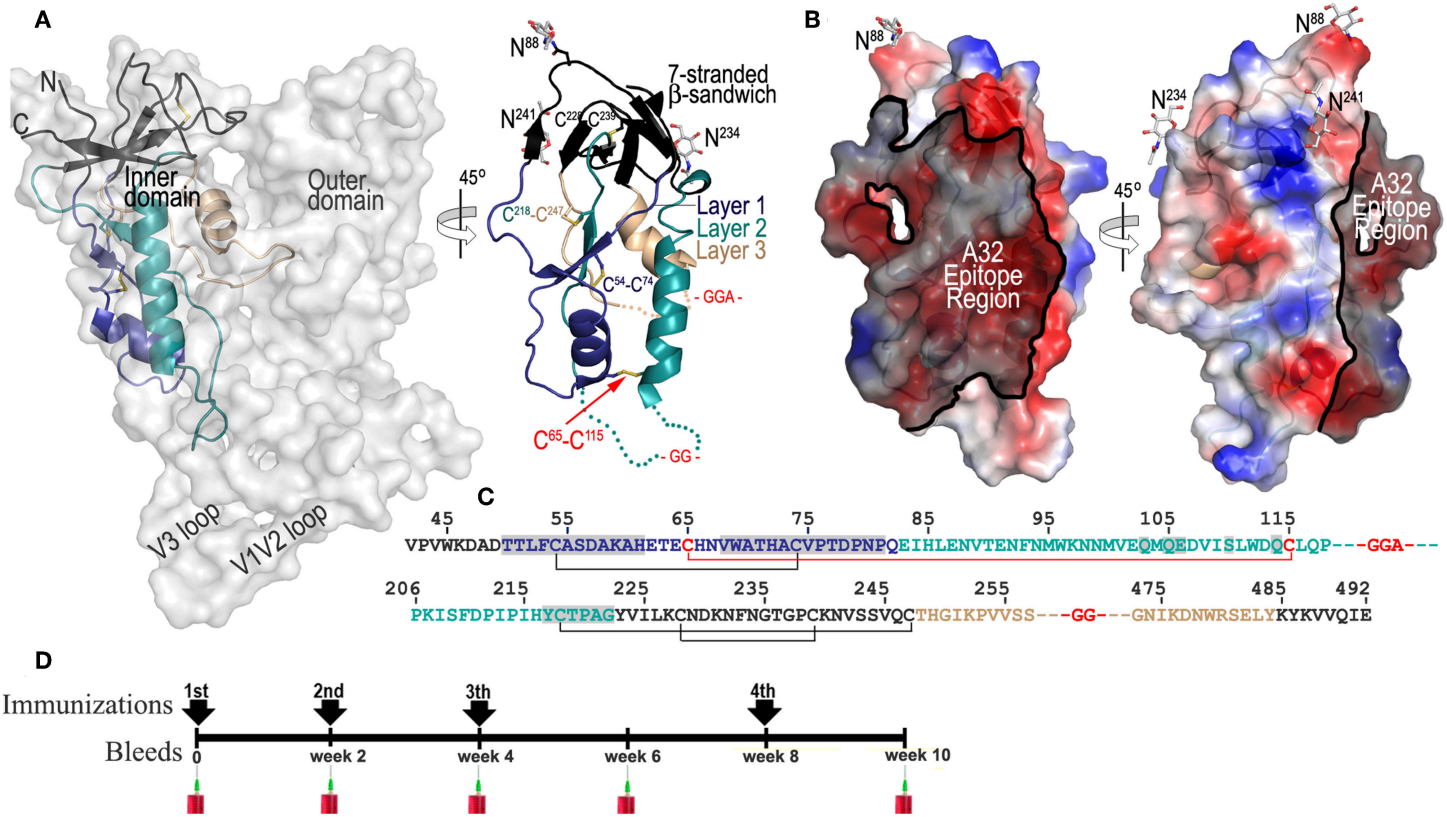
ID2 immunogen design and immunization scheme. **(A)** Putative and crystallographic structure of ID2 immunogen. Putative structure of ID2 is shown overlaid over the structure of full length gp120 from the CD4-triggered BG505 SOSIP trimer (PDB code:1U1F) and a 45° rotation shows the crystal structure of ID2 immunogen from the ID2-Fab A32 complex structure (PDB code:4YC2). “Layered” architecture of gp120 inner domain is shown with the 7-stranded β-sandwich colored black, layer 1 in blue, layer 2 in cyan, layer 3 in light orange. The C^65^-C^115^ disulfide bond introduced to stabilize ID2 in the CD4-bound conformation and GGA(GG)-linkers are shown in red. Sugars at positions 88, 234, and 241 are shown as sticks. The region of ID2 disordered in the ID2-Fab A32 complex are show as broken lines. **(B)** The A32 epitope region in the context of ID2 immunogen. The gp120 residues involved in binding of A32 and A32-like antibodies N5-i5, 2.2c, N60-i3, JR4 are highlighted in black over the ID2 molecule. The molecular surface is displayed over the ID2 molecule and the electrostatic potential is shown as red for negative, blue for positive, and white for apolar. **(C)** Primary sequence of the ID2 construct (from gp120 sequence of clade A/E 93TH057 isolate) with disulfide bonds and –GG—linkers shown. Residues forming the A32 epitope region are highlighted in gray. **(D)** Schematic of immunization protocol.

BALB/c mice were purchased from The Jackson Laboratory and housed in the animal facility managed by BIOQUAL's, Inc., Rockville, MD. The mice were cared for in accordance with the Association for the Assessment and Accreditation of Laboratory Animal Care International (AAALAC) standards and all procedures involving animals were approved by the University Committee on Use and Care of Animals (UCUCA) of BIOQUAL, Inc. 6–8 weeks old BALB/c mice (male and female, 6 animals per group) were immunized at week 0, 2, 4, and 8 via IP injections of 20 μg of ID2 protein in different adjuvants. The control group received ID2 immunogen in PBS and two adjuvants were also trialed; ID2 immunogen in Alum (2% aluminum hydroxide wet gel suspension, InvivoGen, Catalog # vac-alu-250), and ID2 immunogen in GLA-SE adjuvant (stable oil-in water emulsion containing TLR-4 agonist developed by Infectious Disease Research Institute, Catalog # IDRI-GLA-SE, known also under the name EM082). Serum samples were collected prior to immunization and 2 weeks after each immunization according to the scheme shown in Figure 1.

### Detection of Serum Immunoglobulin Specific for ID2

The presence and titers of total IgG, IgG1, IgG2a, IgG2b or IgG2c, IgG3, IgA, and IgM antibodies, specific for ID2 recombinant protein in sera of immunized mice were determined by an Enzyme Linked Immunosorbent Assay (ELISA) using a 100-μL-per-well volume format. Blocking Buffer (Tris-buffered saline (TBS; 10 mM Tris and 100 mM NaCl; pH 8.0) with 5% no fat dry milk and 0.1% Nonidet P-40) was used as blocking solution and as diluting solution for sera and detecting Abs. TBS-T buffer (TBS with 0.1% Tween-20) was used as washing solution. ELISAs were performed as follows: ID2 recombinant protein (0.5 μg/ml) was adsorbed onto ELISA plates (Immunoblot 2HB Thermo, Milford, MA) overnight at 4°C. The plates were washed three times and incubated with 100 μl blocking buffer per well for 2 h at room temperature. Serially diluted sera, beginning at 1:100, were then added and allowed to react with the coated antigen for 2 h at 37°C. Sera was removed, the plates washed, and alkaline phosphatase-conjugated goat anti-mouse IgG (Sigma cat#A3562), IgG1, IgG2a, IgG2b, IgG2c, and IgG3 (SouthernBiotech cat# 1071-04, 1081-04, 1091-04, 1078-04, 1103-04, respectively), IgA and IgM antibodies (SouthernBiotech cat #1040-04 and 1021-04, respectively) diluted 1:1000 in blocking buffer, were added followed by incubation for 1 h at 37°C. After removal of unbound antibody and washing, the Blue Phos Microwell Phosphatase Substrate System (KPL 50–88-00) was used as a substrate to quantitate bound antibody. After 15 min incubation at room temperature, the reaction was stopped using APstop Solution (KPL 50-89-00) and the optical density was read on a microplate reader (SpectraMax Paradigm Multi-Mode Detection Platform Molecular Devices) at 620 nm. The anti-ID2 antibody half-max binding serum titer was calculated using a Microsoft Excel iteration formula.

### Competition ELISAs

To determine if the immunization with ID2 immunogen had induced Cluster A like serum Abs, sera from the terminal bleed were tested for their ability to compete with Cluster A mAbs (A32, N5-i5) for the binding to ID2 in an ELISA setting. Plates were coated o/n at 4°C with 0.5 μg/ml of ID2. Ten-fold serial dilutions of immune sera and pre-immunization sera were mixed 1:1 with biotinylated human CD4i anti-envelope mAb at a concentration correspondent to the half max binding concentrations for each tested mAb; 0.66 μg/mL of A32 and 0.2 μg/ml of N5-i5. The mixtures were then added to previously washed and blocked plates. As a control, a 10-fold dilution of unbiotinylated CD4i mAbs A32 and N5-i5, starting at 10 μg/mL, were tested in the same assay. Sera and Abs were prepared as 2x solutions. After 2 h incubation at r.t. assay wells were washed and incubated with avidin-AP (Invitrogen 1:1,000 dilution) and then with the Blue Phos Microwell Phosphatase Substrate System. Biotinylated-mAb binding was determined by measuring absorbance at 620 nm. Competition percentage was calculated using GraphPad Prism as follows: 0% inhibition was defined as the mean OD value of the lowest serial dilution of the pre-immunization sera (1:10^6^) while 100% inhibition was defined as the mean OD value of the highest concentration tested for unbiotinylated mAbs A32 and N5-i5 (10 μg/ml).

### Sera Reactivity With Denatured ID2 Protein

To assess if immunization with ID2 recombinant protein elicited serum antibodies recognizing conformational epitopes on ID2, pooled sera collected 2 weeks after the last immunization were incubated in solution o/n at 4°C with 1 μg/mL of denatured ID2. ID2 recombinant protein denaturation was performed as previously described in Moore et al. (35), with little modification. Briefly ID2 protein (final concentration 200 μg/ml) was mixed with 10 mM DTT, 0.1% SDS (Sigma-Aldrich), 0.1% FBS (GIBCO-Termo Fisher Scientific), and incubated at 70°C for 10 min. After incubation, the denatured protein was diluted 1:10 in TBS and stored at −20°C until used. The mixtures of denatured protein and pooled sera were then added to a plate coated with ID2. Pooled sera from each group were used as a control. Goat anti-mouse IgG Alkaline Phosphatase conjugated was used as secondary Ab. Plates were read at 620 nM after addition of Blue Phos Microwell Phosphatase Substrate System as previously described.

### Antibody-Dependent Cell-Mediated Cytotoxicity (ADCC)

The ADCC activity of immunoglobulins present in mouse sera collected at week 10 were tested with the optimized rapid fluorometric antibody-dependent cellular cytotoxicity (RFADCC) assay (36). Briefly, EGFP-CEM-NKr-CCR5SNAP cells sensitized with recombinant BaL gp120 were used as targets and human PBMCs were utilized as effectors. Sera were serially diluted three-fold starting at 1:100 through 1:1,968,300 together with control mAbs (N5-i5-positive and Synagis-negative controls). After 2 h of incubation the samples were fixed and collected (at approximately 20,000 events per sample) on a Fortessa Special Order instrument (BD Biosciences) and analyzed using FlowJo software (Tree Star, Ashland, OR). ADCC activity (shown as % cytotoxicity) was defined as the percentage of EGFP-CEM-NKr-CCR5-SNAP target cells that lost GFP staining but retained the CCR5-SNAP tag staining. The results represent the average of the samples tested in triplicate and normalized to the N5-i5 positive control. Max lysis was defined as the maximum percent lysis at any sera concentration. Ec_50_ was determined using a GraphPad prism formula of Log(agonist) vs. Normalized response for a variable slope.

### Neutralization Assay

Mouse sera collected 2 weeks after the 4th immunization and pre-immune sera, were tested in a TZM-bl assay for the presence of neutralizing antibodies. Briefly, 3-fold serial dilution (starting from 1/100 dilution) of sera were mixed with JR-FL pseudo-virus (TCID of 45000 Relative Luminescence Unit) and incubated at 37°C in 5% CO_2_ atmosphere for 60 min at room temperature. TZM-bl cells (10,000/well in complete RPMI with 11 μg/mL DEAE-Dextran) were then added to the sera-pseudo virus mix and plates were incubated for 48 h at 37°C in 5% CO_2_ atmosphere. One hundred and Fifty Microliter of supernatant were removed and 100 μL/well of BrightGlo was then added to each well and after a 2 min incubation to allow complete cell lysis, 100μL from each well was transferred to 96 well black plates. Plates were read with a luminometer using the Promega BrightGlo program. Percent neutralization was determined by calculating the difference in average relative luminescence units (RLU) between virus control (no serum/antibody) and test wells (cells + serum or antibody sample + virus), dividing this result by the difference in average RLU between virus control (cell + virus) and cell only wells, and multiplying by 100.

### Statistical Analysis

Differences in responses between ID2 alone, ID2+Alum, and ID2+GLA-SE were analyzed using a Two-way ANOVA with Bonferroni post-test comparing every sample to every other sample. All statistical analysis was carried out using GraphPad Prism (Version 5 for Windows, San Diego, CA, USA). ^****^represents statistical significance of *P* < 0.0001, ^***^P < 0.001, ^**^P < 0.01, and ^*^P < 0.05. Blue stars represent the difference between ID2 alone and ID2 + Alum, red stars represent differences between ID2 alone and ID2 + GLA-SE and purple stars represent differences between ID2 + Alum and ID2 + GLA-SE.

## Results

### ID2 as an Immunogen Candidate Selectively and Stably Presents the A32 Region

ID2 was designed to stably present the conformational CD4-inducable epitopes of the A32 region within a minimal structural unit of gp120 without any other known (neutralizing or non-neutralizing) epitopes present (34). The design of ID2 was guided by detailed analysis of the epitope structures of A32 and several A32-like antibodies (14, 24, 25) that involve the Env antigen binding residues exclusively within the gp120 inner domain of the constant regions 1 and 2 (C1–C2). Through several steps of structure-guided design we obtained a construct consisting of only 154 residues of the gp120 inner domain which is stabilized in the CD4-bound conformation by the addition of a C_65_-C_115_ disulfide bond (Figures 1A–C). In the ID2 construct the outer domain, variable loops and receptor binding sites were removed to form a minimal structural unit which engrafts only the A32 epitope region. Figures 1A,B show the putative and crystallographic structure of ID2, determined previously in a complex with the Fab of the A32 antibody. ID2 constitutes only one third of the full length gp120 molecule with the A32 epitope region mapping to almost half of the ID2 surface. In addition, a significant area of the ID2 face which does not harbor the A32 epitope is masked by N-glycosylation (asparagines at positions 88, 234, and 241) most likely rendering this part of molecule immunologically silent (Figures 1B,C). We showed previously that ID2 is folded to fully preserve the conformation of the inner domain as seen in the context of CD4-triggered gp120 and stably presents the functional A32 epitopes within the C1–C2 region and thus constitutes a novel immunogen candidate for selective induction of A32-like responses (34).

To evaluate if ID2 indeed is capable of selective induction of humoral response to the desired A32 region we performed immunogenicity studies using the recombinant preparation of ID2 obtained by mammalian cell culture (to preserve wild-type glycosylation) and BALB/c mice as an animal host. Purified ID2 protein, 20 μg per injection, was used to immunize groups of mice (6 animals per group) in the absence of adjuvant (in PBS) and with two adjuvant choices; Alum (2% aluminum hydroxide wet gel suspension) and GLA-SE (a stable oil-in water emulsion containing TLR-4 agonist adjuvant developed by Infectious Disease Research Institute). Immunizations were done according to the immunization scheme shown in Figure 1D with three immunizations in 2-week intervals and a fourth at week 8. Sera was collected 2 weeks after each immunization and analyzed individually for each mouse.

### Immunization With Recombinant ID2 Protein Induced Specific Anti-ID2 Responses in BALB/c Mice

**Figure 2:**
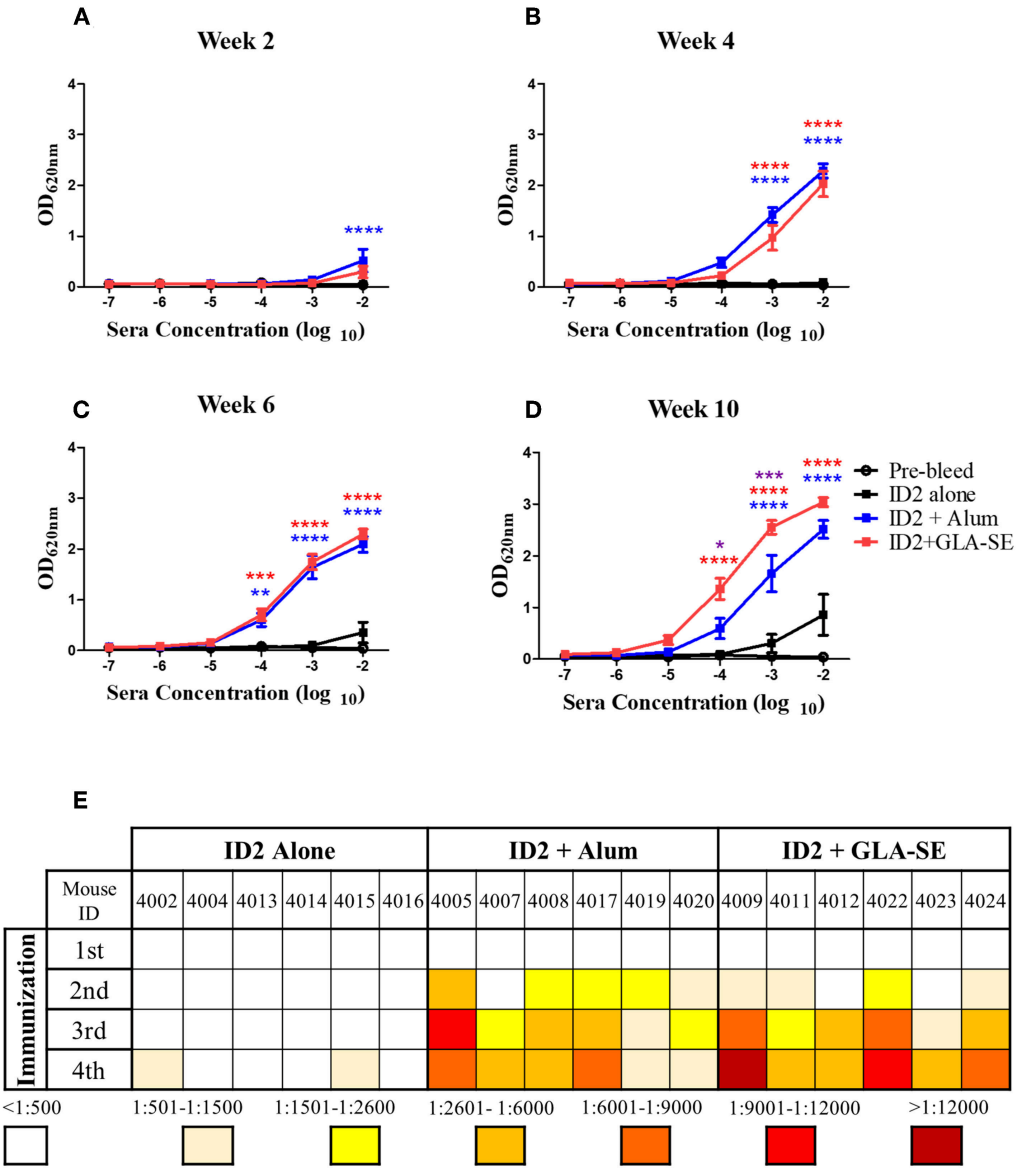
Kinetics and titers of anti-ID2 immune response in immunized mice. Sera collected 2 weeks after each immunization were tested in ELISA for the presence of anti-ID2 specific immune responses (total αID2 IgG). Each sample was assayed in duplicate, displayed is the Mean ± SEM of the 6 mice in each group for **(A)** Week 2, **(B)** Week 4, **(C)** Week 6, and **(D)** Week 10. **(E)** The half-max binding for each individual mouse, identified with a unique number, at each time point following an immunization (1–4) was calculated. *n* = 6 mice for each group. **** represents statistical significance of *P* < 0.0001, ***P < 0.001, **P < 0.01, and *P < 0.05. Blue stars represent the difference between ID2 alone and ID2 + Alum, red stars represent differences between ID2 alone and ID2 + GLA-SE and purple stars represent differences between ID2 + Alum and ID2 + GLA-SE.

Sera collected 2 weeks after each immunization were tested in a sandwich ELISA to assess the presence of specific anti-ID2 serum antibodies. As shown in Figures 2A–D, immunization with ID2 with and without adjuvant elicited an anti-ID2 humoral immune response in all immunized mice, although with significant differences among the 3 immunization groups. Some mice immunized with ID2 without adjuvant developed a weak anti-ID2 specific humoral immune response with a half max binding titers above 1:500, but only after the 4th immunization (Figure 2E). In contrast, titers and kinetics of the humoral immune response was quite different in mice immunized with ID2 delivered alongside Alum or GLA-SE adjuvants. In both groups the immunization induced a detectable specific humoral immune response after the 1st immunization, however, only mice injected with ID2+Alum had higher levels of specific IgG compared to ID2 injected alone (Figure 2A). After 2 and 3 injections, both adjuvants induced significant levels of ID2-specific IgG above mice injected with ID2 alone (Figures 2B,C) and following the final injection, specific IgG levels were significantly higher in the GLA-SE adjuvant group compared to the Alum group (Figure 2D). For mice immunized in GLA-SE, all sera showed an enhancement in the anti-ID2 specific antibody immune response from 1 to 2 logs after each immunization with half-max binding titers ranging from 1:3,400 to 1:36,000 after the 4th immunization (Figures 2D,E). Altogether, these results clearly indicate that recombinant protein ID2 is immunogenic and that such immunogenicity can be improved by administrating the recombinant protein in combination with an adjuvant. As for the adjuvant, our data clearly suggested that mice immunized with ID2 in GLA-SE produced higher titers of anti-ID2 serum antibodies.

### Immunization With ID2 Immunogen Induces an Antibody Response Specific for the Conformational Epitopes Within the A32 Region

**Figure 3:**
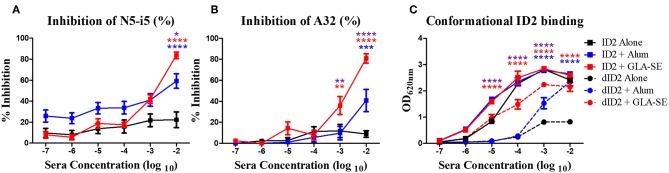
Epitope and conformational specificity of serum antibodies elicited in immunized mice. Sera collected 2 weeks after the final immunization (week 10) were tested for their ability to compete with Cluster A biotinylated mAbs for the binding to ID2 recombinant protein in ELISA. Plates were coated with 0.5 μg/mL of ID2 and serum was incubated with **(A)** A32 and **(B)** N5-i5 (right panel) at half max binding concentrations. Each sample was assayed in duplicate before calculation of % inhibition from the average. **(C)** To assess if immunization with ID2 alone or in combination with adjuvants induced serum antibodies recognizing conformational or linear epitopes different dilutions of pooled sera collected 2 weeks after the last immunization (week 10) were mixed o/n at 4°C with a fixed concentration of denatured ID2. Sera were then reacted in ELISA on ID2 coated plates. Anti-mouse IgG alkaline phosphatase conjugated was used to detect the binding of serum antibodies. **** represents statistical significance of *P* < 0.0001, ***P < 0.001, **P < 0.01 and *P < 0.05. Blue stars represent the difference between ID2 alone and ID2 + Alum, red stars represent differences between ID2 alone and ID2 + GLA-SE and purple stars represent differences between ID2 + Alum and ID2 + GLA-SE.

By design ID2 consists of two faces; a face that harbors the conformational epitopes of the A32 region and a face that is exposed by the removal of the OD which does not have any known epitope targets. Although the epitopes within the newly exposed face are not known, this face might harbor epitopes that are rendered immunodominant by their exposure. To assess if the antibody response induced by ID2 is indeed specific for the desired A32 epitope region we tested sera collected after the final immunization in a competition ELISA with mAb A32 and the A32-like antibody N5-i5 (14). Our assay format was designed to detect if serum antibodies inhibited the binding of mAb A32 or N5-i5 to the recombinant ID2 protein immobilized on microplates. As shown in Figures 3A,B, the immune sera of mice immunized with ID2 alone were not able to inhibit the binding of the tested mAbs to the coated ID2 protein. In contrast, mice immunized either with ID2 in Alum or in GLA-SE elicited antibodies capable of blocking A32 (Figure 3A) and N5-i5 (Figure 3B) at a significantly higher level than sera from mice injected with ID2 alone. Sera from mice immunized with the GLA-SE adjuvant inhibited the binding of over 80% of both N5-i5 and A32, significantly higher than mice injected in the presence of Alum. These data indicate that immunization with ID2 leads to elicitation of a serum antibody response specific for Env targets that overlap with epitopes recognized by the A32 region antibodies A32 and N5-i5 with the adjuvant GLA-SE eliciting the most robust humoral responses.

Next, we asked if the elicited antibody responses were directed toward conformational or linear epitopes within the ID2 immunogen. Pooled sera collected after the final immunization were incubated in solution with denatured ID2 (dID2) protein and then probed in ELISAs with non-denatured ID2 immunogen coated on microplates. As shown in Figure 3C, after being adsorbed with denatured ID2 in solution, sera of all 3 immunization groups were still able to bind the non-denatured ID2. The residual sera of mice immunized with ID2 + GLA-SE adjuvant showed higher levels of binding to conformationally intact ID2 than mice injected with ID2 alone and GLA-SE led to significantly higher levels than alum. This indicates that immunization with ID2 protein alone or in combination with adjuvants leads to elicitation of sera antibodies that recognize and bind conformational ID2 epitopes with a higher titer of conformational antibodies present in sera of mice immunized with the GLA-SE adjuvant.

### Sera of Mice Immunized With ID2 in GLA-SE Mediates ADCC

**Figure 4:**
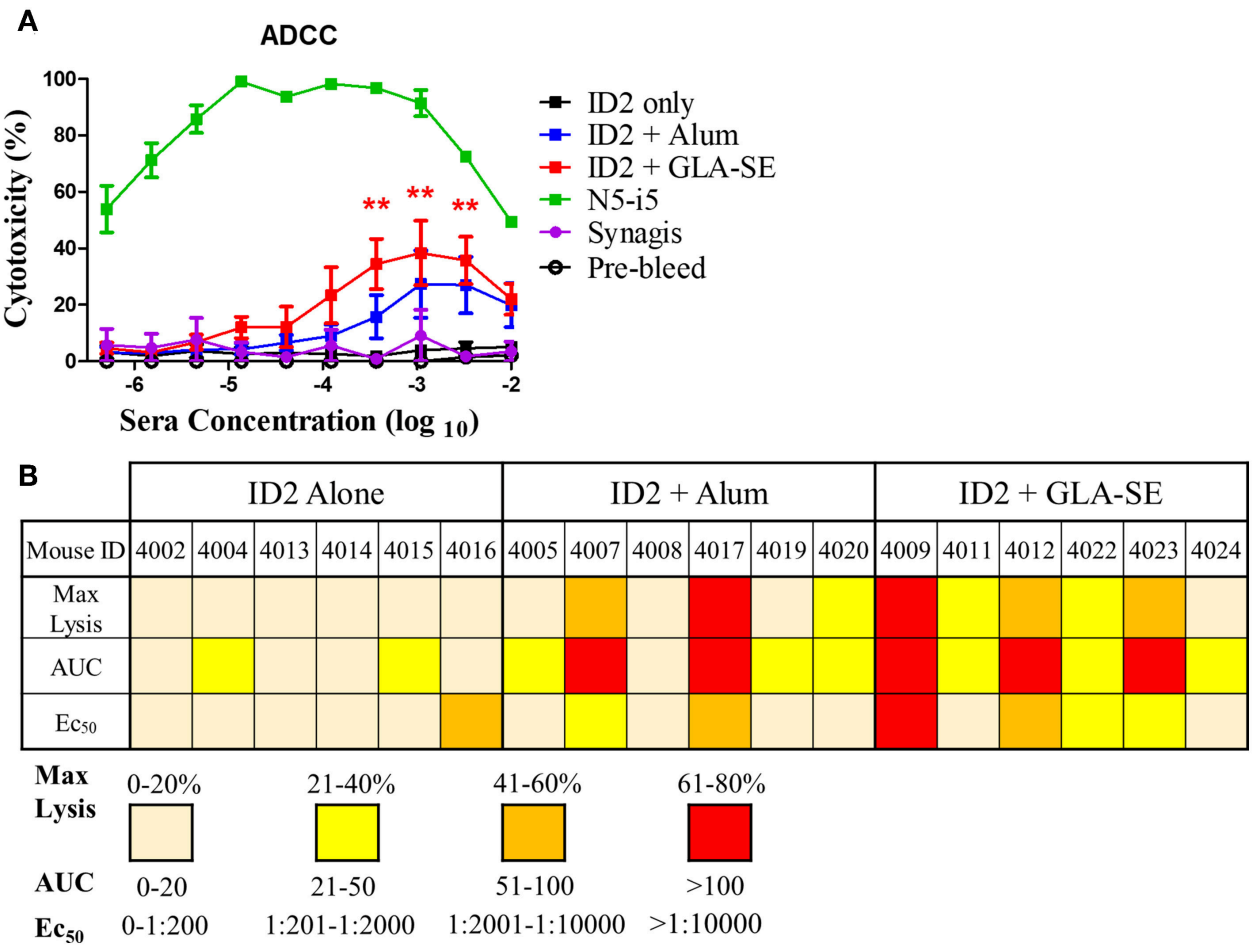
ADCC in sera from mice immunized with ID2 with and without adjuvant. **(A)** Pre-immune sera and sera collected after the last immunization were tested in Rapid Fluorescence ADCC assay against EGFP-CEM-NKr-CCR5SNAP cells sensitized with recombinant BaL gp120 to assess their ability to mediate cytotoxicity. Results are reported as % Cytotoxicity, in relation to maximal cytotoxicity by N5-i5. All samples were analyzed in triplicate before calculating the % cytotoxicity from the mean. No statistical difference existed between ID2+Alum and ID2+GLA-SE at any sera concentration, determined using a Two-way ANOVA test with Bonferroni post-test. **(B)** The maximum lysis (%), Area under the curve (AUC) and Ec_50_ for each individual mouse, identified with a unique number, at each time point following the final immunization was calculated. Max lysis was defined as the maximum percent lysis at any sera concentration. Area under the curve (AUC) was calculated using an Excel formula. Ec_50_ was determined using a GraphPad prism formula of Log(agonist) vs. Normalized response for a variable slope. *N* = 6 for each group. ** represents statistical significance of *P* < 0.01. Red stars represent differences between ID2 alone and ID2 + GLA-SE.

The ID2 immunogen was designed to harbor A32 or A32-like epitopes involved in potent ADCC responses against target cells during the earliest stage of viral entry i.e., at the interaction of gp120 of the Env trimer with the host cell receptor CD4 (14, 21–23, 37) and HIV infected/budding cells which retain CD4 at the target cell surface. Antibodies recognizing the A32 region epitopes were shown to lack conventional neutralizing activities [(12–16, 38), reviewed in (21–23)]. To test if ID2 elicited antibodies capable of ADCC against CD4 inducible (CD4i) targets of a cross clade gp120 we characterized the terminal sera of immunized mice with the optimized RFADCC assay (36) using NKr cells sensitized with recombinant BaL gp120. As shown in Figure 4A, sera from mice immunized with ID2 in the absence of adjuvant showed no ADCC activity, with cytotoxicity readings comparable to the negative control Synagis and the pre-bleed samples. Sera analyzed from mice immunized with ID2 + Alum were capable of modest but not significant ADCC above ID2 alone samples with peaks of cytotoxicity for sera dilutions of 10^2^-10^4^. In contrast, sera from mice injected with ID2 + GLA-SE elicited significant ADCC when compared to ID2 alone (Figures 4A,B).

**Figure 5:**
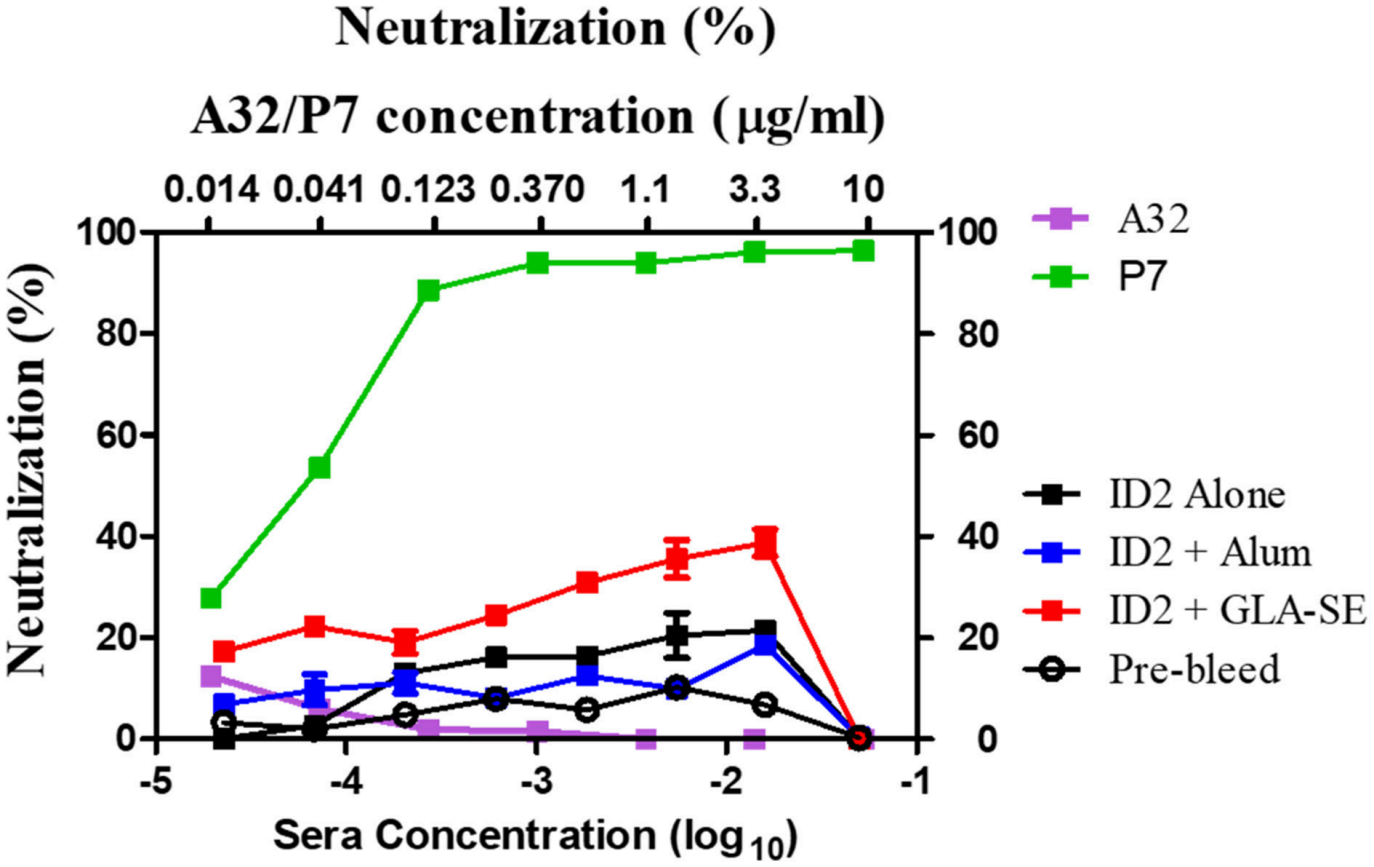
Neutralization in sera from mice immunized with ID2 with and without adjuvant. Sera were tested in a TZM-bl assay for the presence of neutralizing activities against clade B Tier 2 JRFL pseudo-virus. Sera from Pre-bleed (Hollow circle), ID2 Alone (Black square), ID2 + Alum (Blue square), and ID2 + GLA-SE (Red square) were diluted and assayed for their ability to neutralize a clade B Tier 2 JR pseudo-virus (Bottom X axis). These samples were run alongside diluted A32 antibody (orange square) for a negative control and P7 antibody (green square) as a positive control, beginning at 10 μg/ml (Top X axis).

ID2 was designed specifically to contain no known neutralizing epitope targets (34) in order to closely mimic the response observed in the RV144 vaccine trial in which ADCC in the absence of neutralization was associated with protection (28). To test if the ID2 immunized sera contained any neutralizing antibodies we performed a standard neutralization TZM-bl assay against the Tier 2 clade B virus JRFL. This virus was selected to represent a biologically relevant strain for demonstrating neutralization. The recent publication by Montefiori et al. (39) concludes that tier 2 viruses represent the Env conformation of most circulating viruses and are therefore the most appropriate for determining neutralization potential of antibodies. Using sera collected after the last immunization and mAbs A32 and P7 as respective negative and positive controls, we were able to determine that none of the tested sera was able to robustly neutralize the JRFL virus (Figure 5).

### Mice Immunized With ID2 in GLA-SE Show a Larger Diversity of Anti-ID2 Ig Subclasses

**Figure 6:**
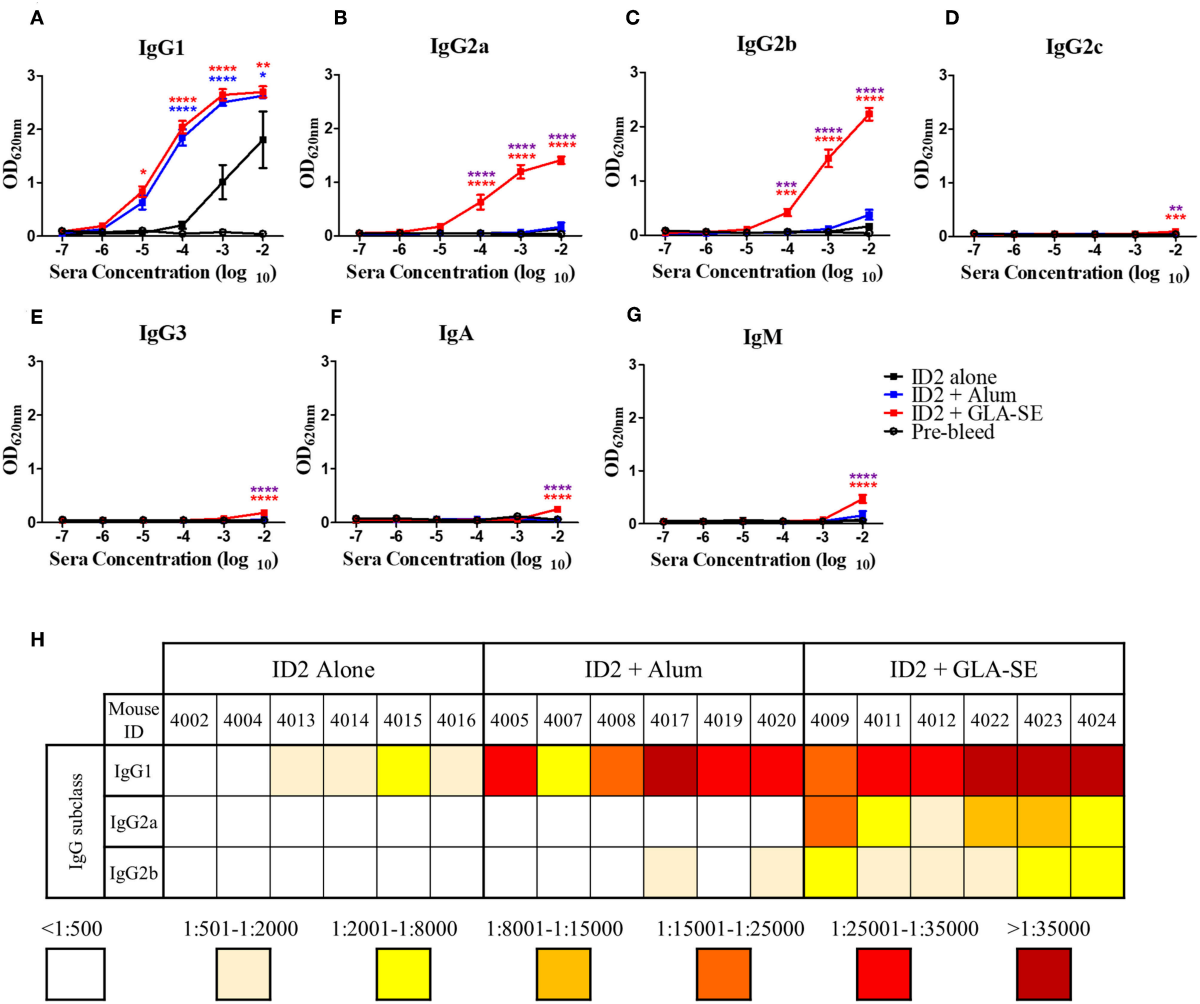
Evaluation of IgG subclasses, IgA and IgM elicited in immunized mice. 10-fold dilutions of pre-immunized (pre-bleed, Black circle) and immunized mice sera collected after last immunization (week 10), were evaluated in ELISA to assess the IgG subtypes; **(A)** IgG1, **(B)** IgG2a, **(C)** IgG2b, **(D)** IgG2c, **(E)** IgG3, as well as **(F)** IgA, and **(G)** IgM induced with no adjuvant (Black square), with Alum (Blue square), or with GLA-SE (Red square). **(H)** The half-max binding for each individual mouse sera sample, identified by an individual number, for the terminal bleed was calculated and displayed for IgG1, IgG2a, and IgG2b. *n* = 6 mice for each group. **** represents statistical significance of *P* < 0.0001, ***P < 0.001, **P < 0.01, and *P < 0.05. Blue stars represent the difference between ID2 alone and ID2 + Alum, red stars represent differences between ID2 alone and ID2 + GLA-SE and purple stars represent differences between ID2 + Alum and ID2 + GLA-SE.

In addition to showing that ADCC in the absence of robust neutralization was adequate for protection against HIV, the RV144 trial also highlighted the importance of the type of antibody repertoire raised. A strong IgG response in the absence of IgA conferred the best protection (28). We therefore aimed to determine the antibody repertoire triggered in response to ID2 in the presence and absence of adjuvants. We tested sera collected after the last immunization (week 10) for the presence of anti-ID2 specific IgG1, IgG2a, IgG2b, IgG2c, IgG3, IgA, and IgM (Figures 6A–G). As expected, the IgG1 subclass was detected in all immunization groups albeit with different titers. Mice immunized with ID2 alone had very low half-max binding titers, ranging between 1:341 and 1:2,344 (Figure 6H). The addition of Alum increased the level of ID2-specific IgG1 with half-max binding titers ranging from 1:7,597 to 1:66,337 in these sera. The half-max binding titers were similar for mice immunized in the presence of GLA-SE, ranging between 1:16,678 and 1:72,864. In contrast, significant differences existed in titers of IgG2a and 2b subclasses between the Alum and GLA-SE adjuvant groups. Mice immunized in the presence of Alum generated a very low titer for both IgG2a and 2b, with half max binding titers for both below 1:500 in all but two mice, while the addition of GLA-SE resulted in half max binding titers for IgG2a ranging between 1:1,833 and 1:20,585 and for IgG2b between 1:572 and 1:3,139 (Figures 5B,C,H). Low levels of anti-ID2 specific IgG2c, IgG3, IgA, and IgM responses were detected only in sera of mice immunized in presence of GLA-SE adjuvant (Figures 6D–G). Combined, these data indicate that immunizations with GLA-SE adjuvant induce a larger diversity of anti-ID2 IgG subclasses as compared to Alum.

## Discussion

Antibodies capable of effective Fc-mediated effector functions, including ADCC, have recently received increasing interest as important components of a vaccine induced humoral response. Although the enthusiasm for this type of antibody function was mostly evoked by the RV144 trial, the evidence also exists from vaccination strategies with Env immunogens in non-human primates (NHP) that link FcR effector functions of antibodies with post-infection control of viremia and/or blocking HIV-1 acquisition, often in the absence of neutralization (40–45), reviewed in (27).

Correlate analyses of the infection risk in the RV144 trial have indicated two gp120 epitope regions; the conformational C1-C2 and the linear V2 loop epitopes, as the major players involved in the Fc-mediated protective response (21, 22, 46). Although only the ADCC response of antibodies directed at the crown of the V2 loop region directly correlated with a lower risk of infection (29–31) the non-neutralizing C1-C2-specific A32-like antibodies synergized with the weakly-neutralizing V2 antibodies (33) to deliver ADCC against neutralization resistant tier 2 isolates. The synergistic crosstalk between antibodies directed at these two epitopes was recognized to be an important component of the protective effect of the RV144 vaccine trial suggesting that C1-C2- and V2-specifc antibodies may act in tandem in a polyfunctional antibody profile to deliver a broad and potent Fc-effector response (33).

Similarly, antibodies specific for Cluster A epitope region (including the A32 subregion) and the co-receptor binding site correlated with sterilizing heterologous protection against SHIV162p3 in NHPs immunized with the conformationally constrained gp120 immunogen, full-length single chain (FLSC) (41, 47). As with the RV144 trial, no correlation between neutralizing activity and protection was observed in these studies pointing again at a role for Fc-mediated effector function in protection against SHIV-1 transmission. In both the RV144 and FLSC trials described above the C1–C2 specific antibodies acted in tandem with antibodies directed at other Env epitopes to contribute to the vaccine efficacy through Fc effector mechanisms. The question remains open if antibodies directed at these non-neutralizing epitope targets could alone afford protection or if they act only as a component of a polyclonal response with antibodies targeting other epitopes. This question has not been addressed, mostly due to the lack of an appropriate immunogen which could selectively bear only this conformational epitope target.

In this study, we investigated the immunogenicity and ability to induce an effective cross-clade ADCC response of a new immunogen candidate ID2, developed previously in our laboratory, as a minimal structural unit of HIV Env stably presenting the non-neutralizing epitopes within the A32 region (C1–C2 epitopes). ID2 consists of the inner domain construct stabilized in CD4-bound conformation by C_65_-C_115_ disulfide bond preserving the A32 region epitopes in the context of CD4-triggered full length gp120, derived from a clade A/E strain, without the complication of any other known neutralizing epitopes. We immunized BALB/c mice intraperitoneally (IP) with 4 doses of ID2 (20 μg /dose) over a period of 8 weeks with three immunizations with 2 weeks intervals and one final immunization delivered 4 weeks later to assess the induction of a memory antibody response. ID2 was administered either alone or with an adjuvant: Alum or GLA-SE. The resulting immune sera were evaluated for the presence of anti-ID2 antibodies (total IgG, IgG subclasses, IgA, and IgM) as well as for their capacity to bind to conformational A32 region epitopes and compete for the binding with mAbs specific for those epitopes. Immune sera were also tested in our RFADCC assay for their ability to mediate ADCC with CEM-NKr-CCR5 target cells sensitized with recombinant gp120 from the Clade B HIV-1_BaL_ isolate and to neutralize a Tier 2 strain of HIV-1. Our results showed that immunization with ID2 alone elicited no or very low anti-ID2 serum antibodies and those elicited were only after the 4th immunization, whereas mice in the groups immunized with ID2 in adjuvants showed robust anti-ID2 responses. Of the two adjuvants, mice immunized with ID2 in GLA-SE exhibited higher anti-ID2 titers at the end of study (week 10) as compared with the Alum group. Interestingly, both groups showed comparable titers after the 3rd immunization, but while in the GLA-SE group the 4th immunization boosted the immune response significantly above the alum group, mice in the Alum group presented a comparable titer to week 6. These data indicate that administration of ID2 in combination with GLA-SE induces enhanced and easily boosted responses to the target antigen. This agrees with the data regarding GLA-SE as an adjuvant, which was designed to promote strong and long lasting T_H_1 responses to protein vaccine antigens (48, 49). Quality analyses of the immune responses elicited by ID2 with adjuvants indicated that antibodies elicited by ID2 with GLA-SE were directed more toward conformational epitopes within the A32 region as shown by the ability of these sera to inhibit the binding of mAb A32 and the A32-like mAb N5-i5 to ID2 and by the residual binding of sera previously incubated with a denatured/linear ID2. Differences were also detected in terms of the IgG isotypes of the elicited antibodies. Mice immunized with ID2 in Alum produced mostly IgG1 antibodies, whereas the immunization with ID2 in GLA-SE induced a broader range and a higher titer of IgG isotypes IgG1, IgG2a, and IgG2b, together with low but detectable IgG3 in addition to IgA and IgM. This difference in antibody isotypes between the two adjuvant groups likely explains the significant higher titers of total IgG in the GLA-SE adjuvant group over the alum group following the final immunization.

ADCC was only significantly increased over the negative control in sera isolated from the GLA-SE adjuvant group, indicating that high titers of ID2-specific mouse IgG1, IgG2a, and IgG2b in addition to small amounts of other isotypes are required for effective RFADCC. In addition to generating more diverse isotypes of ID2 specific antibodies, immunizing with GLA-SE also led to the generation of more specific antibodies – as indicated by significantly higher percent competition for both A32 and N5-i5 antibodies. Interestingly, some sera displayed high ADCC yet low levels of ID2-specific IgG and an absence of class switching from IgG1. One example of this is sample 4,007; when looking at the individual data for the competition ELISA, 4,007 sera inhibits the binding of A32 by 39.7% at the highest sera concentration and N5-i5 by 45%, an average amount for A32 and below average for N5-i5 when compared to other mice in the Alum group. One possibility for a more potent ADCC response is that antibodies to different epitopes were raised in this particular mouse. While the design of ID2 prevents the elicitation of classical C11-like antibodies, some antibodies may be specific for C11-like epitopes which would not have been identified by the competition ELISAs carried out. One known examples of an antibody which could be raised to this area of ID2 is JR4 (24). As previously reported, the RFADCC assay (36) uses antigen sensitized NKr cells and human PBMCs, in which mostly monocyte activity (through their FcγRIIA receptors) is measured. Although interactions between mouse immunoglobulins and human FcγRs are largely understudied there are reports indicating that human FcγRIIA binds mouse IgG1, 2a, and 2b, but not mouse IgG3 (50). All together this suggests that the presence of IgG1 and IgG2a in immunized mouse sera is sufficient to stimulate human PBMCs to mediate the killing of cross-clade antigen sensitized NKr cells.

In conclusion, these results indicate that ID2 can be highly immunogenic, especially when administered together with GLA-SE as an adjuvant, and that the elicited immune response can mediate ADCC in a RFADCC assay using human PBMCs as effector cells. None of the immune sera showed robust neutralization activity against the tier 2 JRFL virus indicating that ID2 as an immunogen induces mostly a humoral immune response against the desired non-neutralizing epitopes within the A32 region. ID2 in combination with GLA-SE was successfully utilized to demonstrate that Fc-effector mechanisms in the absence of strong neutralizing antibodies could be directed toward the A32 subregion which has the potential for HIV-1 protection and ID2 could therefore be utilized for future studies in a NHP challenge study.

## Data Availability

All datasets generated for this study are included in the manuscript and/or the supplementary files.

## Author Contributions

MV, NG, WT, and MP designed, performed research, and analyzed the data. AD, CO, RF, and GL carried out assays and analyzed the ADCC data. RS analyzed the data. MV, RS, and MP wrote the paper. All authors read the manuscript and provided comments or revisions.

### Conflict of Interest Statement

The authors declare that the research was conducted in the absence of any commercial or financial relationships that could be construed as a potential conflict of interest.
